# Cognitive processing characteristics of the advantage of assistant referee offside decision-making: evidence from eye movements and ERPs

**DOI:** 10.3389/fpsyg.2026.1760676

**Published:** 2026-08-03

**Authors:** Shuo Yang, Zhiguang Ji, Chenping Zhang, Liyan Wang, Hongbiao Wang

**Affiliations:** 1 Department of Physical Education, Shanghai University of Medicine and Health Sciences, Shanghai, China; 2 College of Rehabilitation Sciences, Shanghai University of Medicine and Health Sciences, Shanghai, China

**Keywords:** assistant referees, cognitive processing, event-related potential, eye movement, offside decision making

## Abstract

**Background:**

Offside decision-making in football is prone to misjudgments that significantly affect match outcomes. Although video assistant referee (VAR) technology has been introduced, it may cause game interruptions and is subject to technical limitations; thus, improving assistant referees’ on-field judgment is crucial. Previous studies have focused on behavioral differences in refereeing decisions, but the cognitive processing characteristics and neural mechanisms underlying the decision-making advantage of expert assistant referees remain unclear.

**Methods:**

A total of 26 assistant referees were divided into an expert group (level 1 and above, with over 6 years of experience) and a novice group (level 3 and below). A mixed 2 × 2 factorial design (referee level: expert/novice; stimulus type: offside/non-offside images) was used. The stimuli were derived from 2014 World Cup match footage, comprising 25 offside and 75 non-offside images. Eye-tracking technology and a 64-channel event-related potential (ERP) system were used to record behavioral indicators (reaction time and accuracy), eye-movement indicators (number of fixation points, average fixation duration, and gaze trajectory), and ERP components (N2, P300, late slow wave, and PSW) during the decision-making process. Data were analyzed using repeated-measures ANOVA.

**Results:**

(1) Behavioral data: Expert referees responded faster than novices. In offside images, their RT was 2,408.92 ± 276.03 ms. Their accuracy was also higher (0.79 ± 0.10 vs. 0.71 ± 0.13). (2) Eye movement data: Experts made fewer fixations within the region of interest than novices (5.62 ± 1.36 vs. 7.05 ± 1.78). Their average fixation duration was also shorter (220.59 ± 45.69 ms vs. 340.61 ± 118.03 ms). Their attention was primarily focused on the offside line. (3) ERP data: The ERP results were preliminary. Experts showed shorter N2/P300 latencies and lower P300 amplitude. This may indicate faster stimulus evaluation and less additional allocation of cognitive resources. These results should be interpreted cautiously because participant-level artifact-free trial counts could not be reconstructed.

**Conclusion:**

Expert assistant referees showed an advantage in offside decisions. This advantage may be related to a refined visuospatial working memory system. This system enables them to deploy attention quickly, search for key visual cues, and process information efficiently. Behavioral and eye-movement results, together with preliminary ERP evidence, support this interpretation. The findings may provide theoretical support for referee training programs. However, the ERP results should be treated as preliminary because artifact-free trial counts could not be reconstructed.

## 1 Introduction

Misjudgments are common in offside decisions in football. In high-level games, these errors may have a greater influence on match outcomes (Helsen et al., 2006; Kittel et al., 2023; e Pina et al., 2018). In the final stages of the 2002 World Cup, 337 offside decisions were made across 128 games. Incorrect and missed decisions accounted for 26.2% of all offside decisions (Helsen and Bultynck, 2004; Catteeuw et al., 2010c). Up to 88 incorrect offside decisions may have affected match outcomes. This was one reason the 2002 World Cup was highly controversial. Even top assistant referees may make offside errors in important matches. Therefore, it is important to examine the factors that affect offside decisions. It is also important to explore whether training can improve the accuracy of these decisions (Vater et al., 2024; Samuel et al., 2021; Wühr et al., 2020; Wühr and Memmert, 2023; Oudejans et al., 2005; Luis Del Campo and Morenas Martín, 2020).

Video assistant referee (VAR) technology has been used in football to support important decisions, including offside decisions. However, improving the judgment of assistant referees remains necessary (Spitz et al., 2021). Assistant referees must make quick and accurate decisions during a match. This is especially important when VAR cannot provide immediate support. They must understand the rules and respond quickly to changes in play. They also need to communicate well with the referee. VAR can improve decision accuracy, but it may interrupt the game and affect the viewing experience (Lago-Peñas et al., 2021; Das and Daimle, 2022). VAR may also be influenced by technical problems, operational errors, or different interpretations. Accurate on-field judgments by assistant referees can reduce dependence on VAR. They can also help maintain the continuity of the game. Therefore, the professional ability and experience of assistant referees are important for fairness in football (Oliveira et al., 2021). Improving assistant referees’ competence is also important for the long-term development of football in China.

Previous research suggests that assistant referees’ offside decisions depend largely on visual processing of complex pitch information (Catteeuw et al., 2009a). Many studies have used the visual search paradigm to examine perceptual-cognitive expertise in sport decision-making. (Catteeuw et al., 2009b, Catteeuw et al., 2010a) studied assistant referees of different levels by using eye-movement recording. They found that national-level assistant referees made more errors than international-level assistant referees. They also found that referees primarily focused on the relative positions of attacking and defending players around the offside line. This indicates that referees’ attention is closely related to the offside line rather than only to the passing or receiving action. In general, experienced participants are better than novices at extracting perceptual cues. Their higher accuracy and faster responses support this view (Wang et al., 2024).

Expertise-related differences in visual attention have also been found in other sports. In rifle and pistol shooting, experts usually show more efficient cortical activation and more stable gaze behavior than novices. Longer quiet-eye duration is also related to better performance and higher task complexity (Janelle et al., 2000; Williams et al., 2002; Shao et al., 2020; Güler et al., 2023; Bayram et al., 2024). These findings show that expert advantage is not limited to football refereeing. Skilled performers can allocate attention efficiently. They can also stabilize gaze on key information and integrate visual cues under time pressure.

Visual–spatial working memory is an important part of working memory. It temporarily stores and processes spatial information, such as object location and orientation (Baddeley, 2000). The football offside decision task is a typical visuospatial working memory task. In this task, the assistant referee must process the positions of the foremost attacker, the rearmost defender, and the ball at the moment of passing. The goalkeeper and other players may also need to be considered. Therefore, accurate judgment requires useful cue extraction and effective visual search. Visuospatial working memory is primarily associated with the occipital-parietal pathway (van Asselen et al., 2006). Event-related potential (ERP) studies also show strong effects in frontal–parietal regions during visuospatial working memory tasks (Hau, 2012). Offside decisions are made under time pressure. Thus, the speed of cognitive processing is very important. ERPs can help reveal the neural processes underlying spatiotemporal cognition in sports tasks. They can also help explain the neural basis of expertise advantage (Aglioti et al., 2008).

Recent technological advances provide new methods for studying perceptual-cognitive processes in more realistic sport environments. For example, AI-based computer vision can be combined with wearable eye tracking. This method can process gaze data during live games. It can also detect players or events in the scene video and build dynamic areas of interest. This is different from manually defined static AOIs. Lozzi et al. (2025) used two pre-trained AI models and a wearable eye tracker to analyze gaze behavior in live basketball games. This study shows the potential value of AI-assisted gaze analysis. At present, such methods cannot replace controlled laboratory studies. However, they can help future research connect gaze behavior, game context, and decision-making. Therefore, the present study should be viewed as a controlled first step. Future studies should test these findings in dynamic or field-based tasks.

Most previous studies focused on errors and omissions among assistant referees. Some studies also examined the effects of physical function and emotion on decision errors (Helsen and Bultynck, 2004). The performance level of referees also varies across studies. It may range from the international level to the national or regional level. Furthermore, many offside situations are ambiguous and require subjective judgment based on several criteria (Helsen et al., 2023). Therefore, assistant referees may be less accurate in offside situations than in situations with clearer criteria (Put et al., 2016). This usually reflects perceptual processing. The present study used eye movement and ERP techniques to examine the cognitive processing of assistant referees during offside decisions. It focused on visual working memory, visual search, and neural mechanisms in expert and novice referees. The aim was to extend existing theories of sport decision-making. The study also aimed to provide evidence for referee training and learning.

## 2 Methods

### 2.1 Participants

The assistant referees across different football levels were divided into groups: the assistant referees of level 1 and above formed the expert group (mean age 35.46 ± 3.64), and those at level 3 and below formed the novice group (mean age 25.21 ± 3.95). Thirteen subjects in each group participated in the experiment; the expert group subjects were primarily from the Liaoning Provincial Football Association, Shenyang Municipal Football Association, and Changchun Municipal Football Association. Eight of them were currently assistant referees in the Chinese Football Association Super League (CFA Super League), and five had enforced the law in the first and second divisions of the Chinese Football Association. The total enforcement experience of the expert group exceeded 6 years. The test subjects in the novice group were all graduates and students of a sports university specializing in football and had served as assistant referees with some prior experience. All experimental participants were disease-free, had no family history of genetic diseases, no psychological or neurological disorders, no history of brain injuries or other diseases, had normal or corrected-to-normal vision in both eyes, and were not left-handed. None of the participants had participated in such an experiment before the experiment began, and they received compensation afterward. The study was approved by the Shenyang University of Sport Ethics Committee.

### 2.2 Experimental design

The experiment used a mixed 2 × 2 two-factor design with two independent variables. Factor 1-Referee level was the between-group variable and was divided into two levels: expert and novice. Factor 2, stimulus material type, was the within-group variable and was divided into offside and non-offside images. The dependent variables were behavioral indicators, including reaction time (RT) and accuracy (ACC) of participants; eye movement recorder to record the number of participants ‘gaze points in the area of interest, the duration of the first gaze point, the average gaze duration, eye movement trajectories, and hotspot maps; and indicators of the event-related potentials of brain neuronal activity, including latency and amplitude.

### 2.3 Stimulus material

All footage was edited from videos of the 2014 FIFA World Cup in Brazil (64 games in total). This was done to make the stimulus materials as homogeneous as possible. A national referee used Color Shadow 10.0 to edit the videos. Images of offside and suspected offside situations were then intercepted. Each image was captured at the moment when the ball left the attacking player’s body (head or foot). At that moment, a teammate was in an offside or non-offside position. Three national assistant referees independently rated the suitability of each image on a 1–9 scale. A score of 1 meant unsuitable, and 9 meant suitable. They also rated the difficulty of each image on a 1–9 scale. A score of 1 meant too easy, and 9 meant too difficult. Images with proper suitability and difficulty were retained. Disagreements were discussed until a consensus was reached. Finally, 25 offside images and 75 non-offside images were selected.

### 2.4 Experimental equipment

The ERP equipment was a 64-channel ERP recording and analysis system from NeuroScan (USA). The system used an international 10–20 extended 64-channel electrode cap provided by Feiyuxing Technology Co., Ltd. The electrodes were made of Ag/AgCl. The reference electrodes were placed on the mastoids behind both ears. Horizontal and vertical electro-oculographic electrodes (EOG) were placed near the right eye. They were attached to the lateral and lower sides of the eye, respectively, about 1 cm from the eye. After the electrodes were fixed, conductive paste was injected into each electrode site. The paste was medical conductive paste GT-5 from Wuhan Greentech Technology Co., Ltd. The impedance of all electrodes was kept below 5 kΩ. The sampling frequency was 500 Hz/channel.

The eye movement experimental equipment was the SMIRED500 remote-controlled desktop oculomotor device manufactured by SMI (Germany), which uses pupillary and corneal reflex points to track eye movements. The sampling rate was 250/500 Hz, the tracking resolution was 0.03° pupil/CR, the accuracy of eye tracking was 0.4°, the distance of the subject from the eye tracker was 60–80 cm, and the distance of the subject was 70 cm. The tracking area of the head was 40 cm × 20 cm. While the ERP recording system was operational, synchronous recording of eye movements and ERP data was realized using E-Prime stimulus presentation and recording software. ERP data.

Two DELL desktop computers with a CPU frequency of 4.0 GHz and Windows 10 were used in the experiments. The monitors were three 19-inch flat-panel displays with a resolution of 1,024 × 768 and a refresh rate of 100 Hz, two of which were used for EEG data acquisition and for presenting stimulus materials, and one was used to observe the subjects. Another 15.6-inch Dell laptop was used to collect eye movement data.

### 2.5 Experimental procedures

The experimental program was created using E-prime 3.0 software, and the experimental paradigm was the oddball paradigm, in which participants were randomly presented with 25 offside images and 75 non-offside images, with each stimulus occurring twice, for a total of 200 trials over approximately 25 min. The participants randomly pressed a button to initiate the experiment. First, the following instruction appeared on the screen: “You are viewing images of a soccer match divided into two categories: offside images and non-offside images. Please judge this as quickly and accurately as possible.” Press “F” if you believe it is an offside situation and press “J” if you believe it is not an offside situation. When you are ready, press any key to begin the experiment. The participants were randomly presented with two types of experimental material.

**Figure 1:**
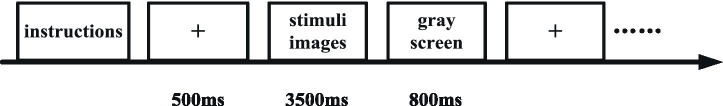
Flowchart of the experiment.

The experiment was carried out in an EEG laboratory at a sports university. The laboratory was clean and quiet. The lighting was soft and diffuse. Participants were contacted one day before the experiment. The purpose was to confirm that they were not unwell and had not consumed alcohol. They were asked to rest before 10:30 p.m. and wake up at 8:30 a.m. This helped maintain good EEG recording conditions. Before the experiment, all procedures were explained to the participants. They then completed the consent form and the basic information questionnaire. The participants washed their scalp with shampoo and dried their hair. They also turned off all electronic communication devices. This was done to reduce electromagnetic interference. Each participant wore an EEG cap. Conductive paste was injected into each electrode site to keep impedance below 5 kΩ. The participants sat at the experimental table. The seat height was adjusted so that the eyes looked at the center of the screen. The jaw was fixed on a U-shaped holder. The eyes were about half an arm’s length from the screen. Participants completed the task independently in the experimental room. The experimenter monitored the process and task quality through the main monitor. The experimental flow is shown in Figure 1. Each participant completed 200 trials.

### 2.6 Statistical analysis

During the experiment, behavioral data, such as correct response time (RT) and accuracy (ACC), were collected and processed using E-Prime software. Eye movement data were recorded using an eye-tracking device and automatically generated in an eye-tracking file, which was matched to the stimulus material using the B-Gaze analysis software. The area of interest selected in this study was the area encircled by two parallel lines between the ball and the offside line. The ERPs data were recorded using an ERPs system manufactured by Neuroscan, Inc. (USA). The data were processed offline using the CURRY8 software, and in the analysis, the connection between the bilateral postauricular mastoids was used as a reference. After ocular artifact removal, EEGs were segmented, filtered, and baseline corrected, and overall averaging was performed (Luck, 2014).

According to the aim of the study, the analysis of the following three segments of the time course was completed: The first segment recorded the image stimulus presentation moment as point 0, intercepting from −200 to 800 ms, analyzing the early ERPS components of the stimulus presentation from 0 to 800 ms, and statistical analysis of their wave peaks and latencies. Segment 2: Using the image stimulus presentation moment as point 0, sections from 800 to 2,500 ms were acquired to observe the late component, and the average wave amplitude was statistically analyzed. Segment 3: Based on a previous study (Hack et al., 2009), the subject’s moment of key press response (offset) was used as point 0, and segments from −200 to 800 ms were recorded to capture the ERPS components of this period and to statistically analyze the wave amplitude and latency. All segments were set to 200 ms (−200 to 0 ms) before the start of the video. All wave amplitude and latency data were analyzed and processed in conjunction with the topographic maps of this study, as well as the waveform maps of the following electrode points: frontal zone (FZ), central zone (CZ), parietal zone (PZ), and occipital zone (OZ). These data were selected for analysis.

Each participant completed 50 offside trials and 150 non-offside trials before artifact rejection. Participants with excessive EEG drift were excluded from ERP analyses. Thus, nine participants remained in each group. The original CURRY/EEG preprocessing logs and participant-level trial-retention records are no longer available. Therefore, the exact number of artifact-free trials for each participant and condition cannot be reconstructed accurately. For this reason, no *post hoc* trial-count values are reported. This limitation is stated in the Limitations section. The ERP findings should be interpreted with caution, particularly for the offside condition and the later P300 and PSW components.

All data were analyzed using repeated-measures ANOVA with SPSS 22.0. Statistics that failed the sphericity test were corrected for degrees of freedom and *p*-values using the Greenhouse–Geisser method, and significant interactions were analyzed using a simple effects analysis. Further discussion of the data was conducted using a statistical threshold of *p* < 0.05.

Partial eta squared (*η*^2^) was used as the effect-size index. We used conventional benchmarks for interpretation: about 0.01 for small effects, 0.06 for medium effects, and 0.14 for large effects. The interpretation was also considered in light of the study context.

## 3 Results

Behavioral data were screened to remove mean values that fell more than three standard deviations from the correct behavioral RT and ACC values. Complete behavioral and eye movement data were available for all 13 participants. Subjects with excessive drift were removed during processing of the event-related potential data, yielding data for nine participants in each group.

### 3.1 Behavioral data

A repeated-measures ANOVA on behavioral outcomes, with referee level (between-subjects variable) and stimulus material type (within-subjects variable) as independent variables and correct RT as the dependent variable, revealed significant differences in correct RT between offside and non-offside. *F*(1,24) = 12.94, *p* < 0.001, *η*^2^ = 0.35, and faster RT in offside contexts. The main effect of group was significant, *F*(1,24) = 4.83, *p* = 0.04 < 0.05, *η*^2^ = 0.17, and there was a significant difference between the expert and novice groups, with participants in the expert group reacting significantly faster than those in the novice group. The interaction effect between stimulus material type and groups was significant, *F*(1,24) = 4.42, *p* = 0.046 < 0.05, *η*^2^ = 0.16. Another simple effect analysis showed that participants in the expert group had significantly shorter decision times for offside images (2,408.92 ± 276.03) than for non-offside images (2,610.03 ± 266.90). However, there was no significant difference in the judgment time of the novice group for the different picture stimuli.

Similarly, a repeated measures ANOVA on the ACC of the behavioral scores revealed that there was significant variability in the ACC for offside versus non-offside, with a significant main effect of offside versus non-offside *F*(1,24) = 6.08, *p* = 0.02 < 0.05, *η*^2^ = 0.20, and that the ACC of the offside images was lower than that of the non-offside images for both experts and novices. There was a significant main effect of group, *F*(1,24) = 4.90, *p* = 0.04 < 0.05, *η*^2^ = 0.17, and the expert group’s ACC (0.79 ± 0.10) was significantly higher than that of the novice group (0.71 ± 0.13). There was a non-significant interaction effect of offside/non-offside with group, *F*(1,24) = 1.33, *p* = 0.26 > 0.05, *η*^2^ = 0.05,

### 3.2 Eye movement data

The eye movement data of all participants in both the offside and non-offside conditions were analyzed with the following results: the results of the repeated measures ANOVA with the duration of the first gaze point in the region of interest as the dependent variable and the group and stimulus type as the independent variables indicated that the difference in the main effect was not significant, and there was no interaction effect between the two variables.

A repeated-measures ANOVA with the number of gaze points in the region of interest as the dependent variable and group and stimulus type as independent variables revealed a non-significant main effect of stimulus type, *F*(1, 24) = 0.07, *p* = 0.80 > 0.05, *η^2^* = 0.03, and a significant main effect of group *F*(1, 24) = 5.31, *p* = 0.03 < 0.05, *η^2^* = 0.22, regardless of whether the offside or non-offside context was the same as the offside context. In our non-offside context, the number of annotation points was smaller in the expert group (5.62 ± 1.36) than in the novice group (7.05 ± 1.78). The interaction was not significant [*F*(1, 24) = 0.1, *p* = 0.77 > 0.05, *η*^2^ = 0.05].

A repeated-measures ANOVA with mean gaze time in the region of interest as the dependent variable and group and stimulus type as the independent variables showed a significant main effect of stimulus type, *F*(1, 24) = 23.20, *p* < 0.001, *η*^2^ = 0.55. The offside mean gaze time was significantly lower than the non-offside mean gaze times for both experts and novices. The group main effect was significant, *F*(1, 24) = 16.45, *p* = 0.001 < 0.05, *η*^2^ = 0.46. The mean gaze time was significantly lower in the expert group (220.59 ± 45.69) than in the novice group (340.61 ± 118.03). The interaction between the two factors was significant [*F*(1, 24) = 17.33, *p* = 0.001 < 0.05, *η*^2^ = 0.48]. Further simple effects analyses showed that the mean gaze duration in the region of interest was significantly higher in the novice group (465.20 ± 141.73) than in the expert group (229.66 ± 47.81) for non-offside contextual judgments. The mean gaze duration within the region of interest of the expert group (211.52 ± 43.56) was also lower than that of the novice group (216.01 ± 94.33) in the offside contextual judgment, but did not reach a significant level.

### 3.3 ERPs data

In this study, the cognitive processing of the participants’ brain to complete the task is divided into three parts: the first part (early component) is the initial process of the task, the preliminary recognition process of the external stimulus; the second part (late slow wave) is the brain’s further analysis and processing of the experimental task, including the process of detecting, perceiving, remembering, analyzing, and judging the experimental materials. The third part summarizes the brain’s previous analysis and judgment processes, which are the main stage of decision-making. Accordingly, the ERPs were segmented as follows: the early component of the first segment assumes the appearance of the stimulus as point 0 (−200 to 800 ms); the second segment of the late slow wave with the stimulus appearing as point 0 (800 to 2,500 ms); the third segment decision-making phase with a response as point 0 (−200 to 800 ms), and the statistics of each time course are as follows:

#### 3.3.1 Early ERP components

**Figure 2:**
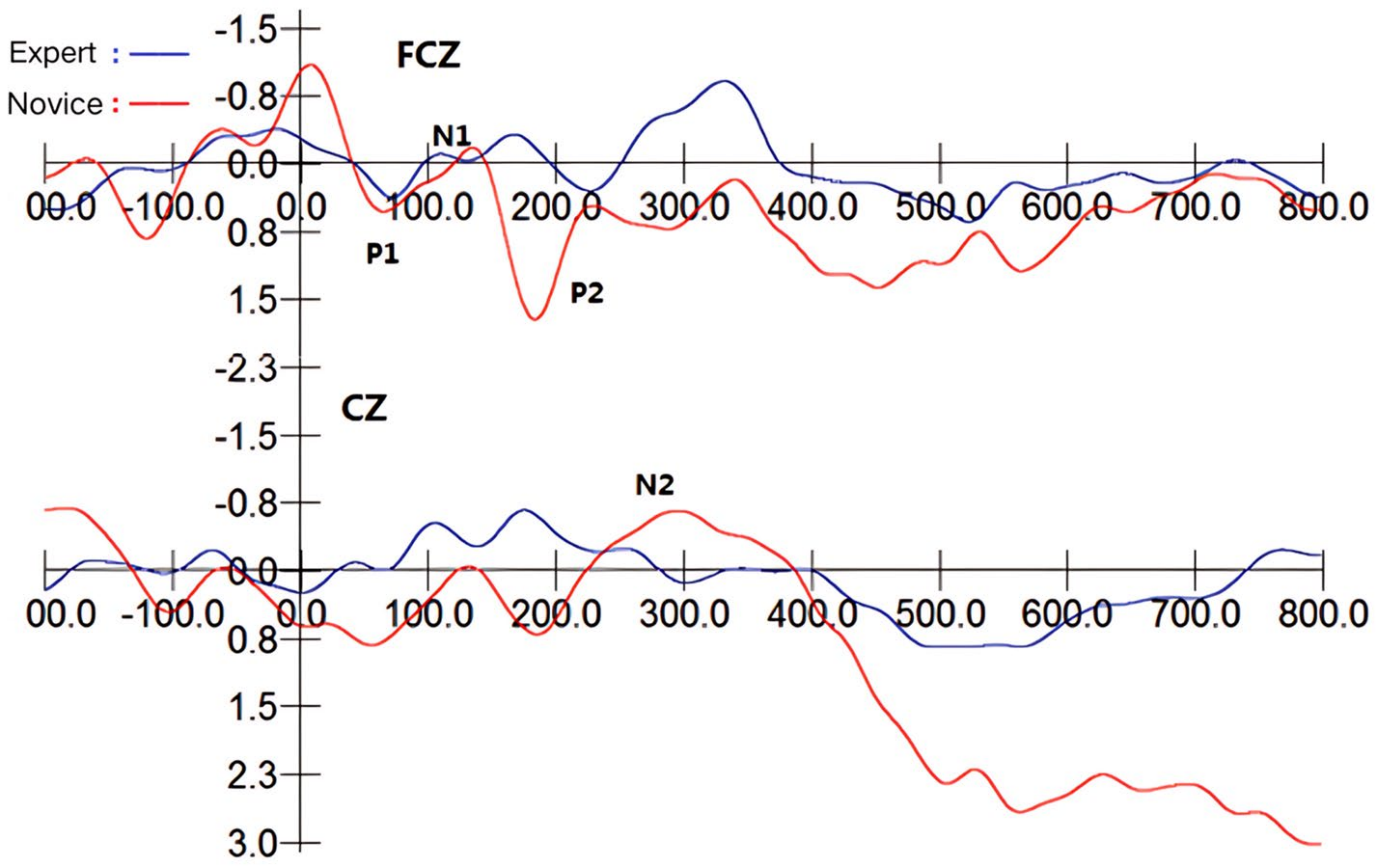
Grand-average ERP waveforms at selected electrode sites for offside decisions in experts and novices.

The observation and analysis of the overall average ERP waveform maps revealed that the two groups of referees with different scores exhibited distinct activation levels in their respective brain regions at approximately 420 ms after the presentation of offside or non-offside images, and the P1, N2, and P300 components were induced. Their properties are shown in Figure 2, where an upward negative wave was induced at each electrode point approximately 250–350 ms after stimulus presentation, which was identified as the N2 component according to the rules for naming and determining time-dependent potential components. According to the regional maps of each electrode point, it was found that it was located in the part of the connection between the frontal zone (FZ) and the central zone (CZ), that is, the frontal central zone (FCZ), and more pronounced wave amplitudes were found in the middle of the parietal zone (PO) and the occipital zone (OZ) of the participants.

According to previous studies (Patel and Azzam, 2005), in many experiments, N200 was found to occur mainly in the front part of the brain, such as the prefrontal region, while in some studies on visual attention, N200 appeared in the front part of the brain rather than the back part of the brain, such as the occipital lobe, where the visual search paradigm is more evident (Folstein and Van Petten, 2008).

In connection with the purpose of this study, event-related potential data from four prominent brain regions of the subject’s head, namely frontal (FZ), central (CZ), parietal (PZ), and occipital (OZ), were selected and analyzed by repeated measures ANOVA using SPSS 22.0 for the two dependent variables, latency and amplitude of the waves.

A repeated-measures ANOVA with the N2 component latency in the central zone (CZ) as the dependent variable and group and stimulus type (offside and non-offside images) as the independent variables showed a significant main effect of group, *F*(1.16) = 7.52, *p* < 0.05, *η*^2^ = 0.32. In the CZ, N2 latency was significantly lower in the expert group (198.95 ± 49.53) than in the novice group (237.45 ± 21.09). There was a significant main effect of stimulus type, *F*(1,16) = 5.02, *p* < 0.05, *η*^2^ = 0.24. In the central zone, the N2 latency in the offside image was significantly lower (204.56 ± 27.55) than that in the non-offside image (231.84 ± 43.07). No interactions were observed between these two factors.

A repeated-measures ANOVA with N2 component latency in the frontal zone (FZ) as the dependent variable and group and stimulus type (offside and non-offside images) as independent variables showed no significant effect on latency in the frontal zone.

A repeated measures ANOVA with N2 component latency in the parietal zone (PZ) as the dependent variable, group with stimulus type (offside and non-offside images) as the independent variable, showed a significant main effect of group *F*(1,16) = 12.59, *p* < 0.001 (CI: 16.55–65.67) and *η*^2^ = 0.44. The N2 latency of the expert group (207.00 ± 23.43) was significantly lower than that of the novice group (230.78 ± 29.41). The interaction between group and stimulus type was significant, *F*(1,16) = 5.07, *p* = 0.04 < 0.05, *η*^2^ = 0.24, and further simple effects analyses revealed that the expert group (186.44 ± 23.25) had significantly lower N2 component latencies than the novice group (228.67 ± 26.39) for context-related offside judgments. When it came to contextual judgment without offside, the experts were also lower than the novices, but the difference was not significant.

A repeated measures ANOVA with occipital zone (OZ) N2 component latency as the dependent variable and group and stimulus type (offside and non-offside images) as independent variables showed a significant difference in the interaction between group and stimulus type, *F*(1.16) = 4.332, *p* < 0.05, *η*^2^ = 0.213. A simple-effects analysis showed that the occipital zone (OZ) of the participants in the expert group was significantly lower than that of the novices in the offside context. The latency (198.00 ± 11.01) was lower than that in the non-violation context (252.92 ± 13.52).

No significant effects were observed in the four brain regions when N2 amplitude was used as the dependent variable.

**Figure 3:**
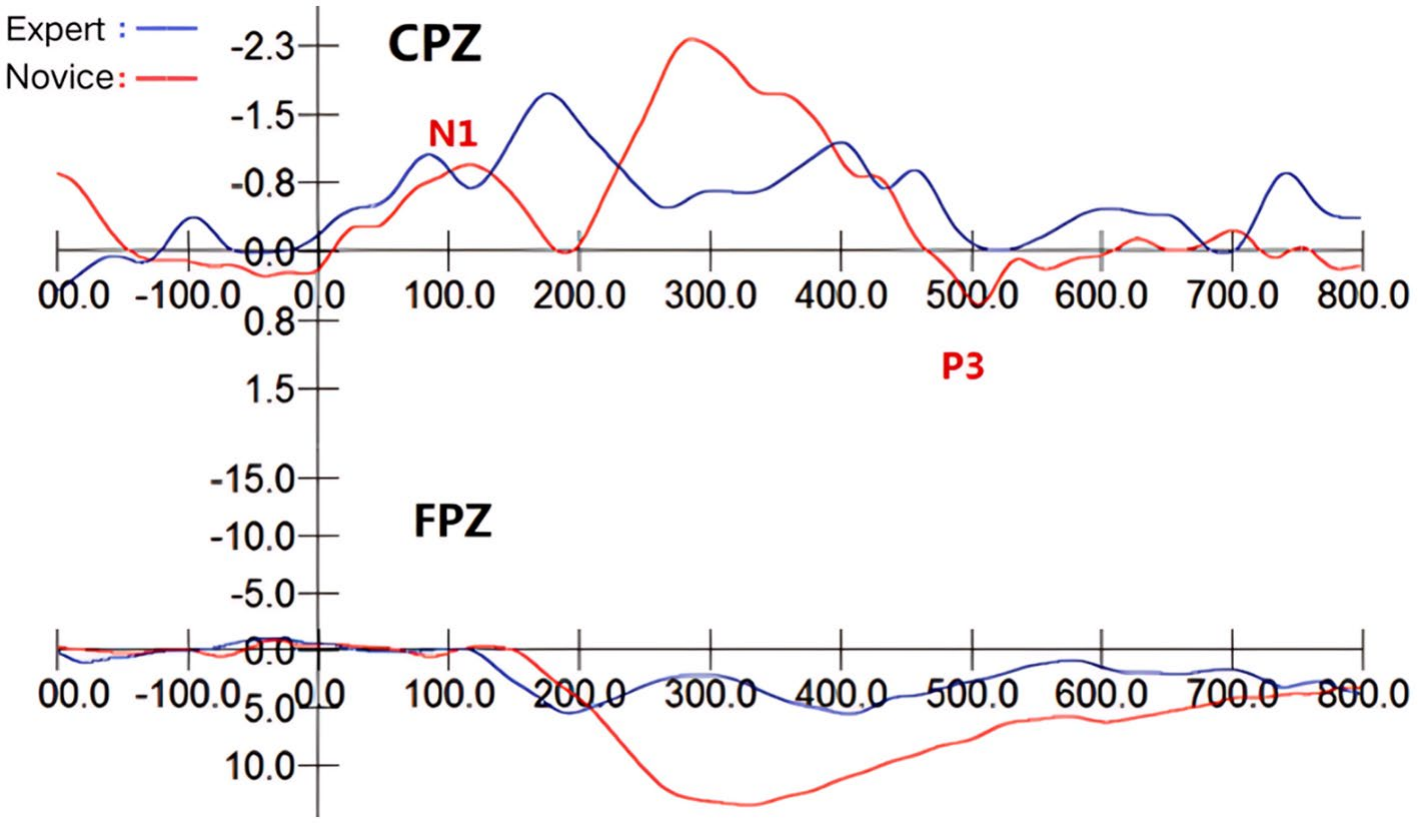
Grand-average ERP waveforms at selected electrode sites for non-offside decisions in experts and novices.

As shown in Figure 3, approximately 300–450 ms immediately after the stimulus appeared, the stimulus elicited a significant downward movement of a positive component, the P300 component, in participants whose advanced ability to anticipate actions is thought to be related to the improved ability, as well as the delayed P2 effects of P300 distribution in the occipital-parietal and posterior regions. Accordingly, in conjunction with the purpose of the experiment on the experimentally generated P300 component, frontal (FZ), central (CZ), parietal (PZ), and occipital (OZ) regions were selected. A repeated-measures ANOVA was performed on the four brain regions with P300 Latency as the dependent variable and group × stimulus type as the independent variable. The results showed no significant difference in latency between the frontal and central zones.

The main effect of stimulus type in the parietal zone (PZ) was not significant [*F*(1, 16) = 0.14, *p* > 0.05, *η*^2^ = 0.01]. The main effect of group was significant, *F*(1,16) = 58.15, *p* < 0.05, *η*^2^ = 0.78. The subjects in the expert group (361.78 ± 54.16) had significantly lower latencies than those in the novice group (511.88 ± 69.96). The interaction was significant, *F*(1,16) = 5.88, *p* = 0.028 < 0.05, *η*^2^ = 0.27. Further simple-effect analyses revealed that the P300 latencies in the novice group were larger in the offside context (542.22 ± 51.97) than in the non-offside context (481.56 ± 79.94).

A repeated-measures ANOVA of occipital zone (OZ) P300 component latencies revealed a significant group main effect, *F*(1,16) = 24.57, *p* < 0.001, *η*^2^ = 0.61. The occipital zone (OZ) P300 component, latency, stimulus type, and main effect were not significant: *F*(1,16) = 0.27, *p* = 0.61 > 0.05, *η*^2^ = 0.02. Interactions marginally significant *F*(1,16) = 4.28, *p* = 0.05, *η*^2^ = 0.211. Another simple effect analysis showed that the occipital P300 latency when assessing the offside images was significantly lower in the occipital area of the expert group (310.22 ± 42.79) than in the novice group (537.44 ± 114.82). However, this difference was not significant in the non-offside contexts.

A repeated-measures ANOVA was performed across groups with different brain regions, with P300 latency as the dependent variable. The results are shown in Table 1.

**Table 1:** Descriptive statistics of the latency of the P300 component in different groups and brain regions in offside situations.

Brain area	Group	n	M	SD
CZ	Novice	9	556.33	61.03
	Expert	9	341.44	63.32
FZ	Novice	9	445.11	115.30
	Expert	9	427.56	125.25
PZ	Novice	9	542.22	51.97
	Expert	9	339.56	38.74
OZ	Novice	9	537.44	114.82
	Expert	9	310.22	42.79

**Figure 4:**
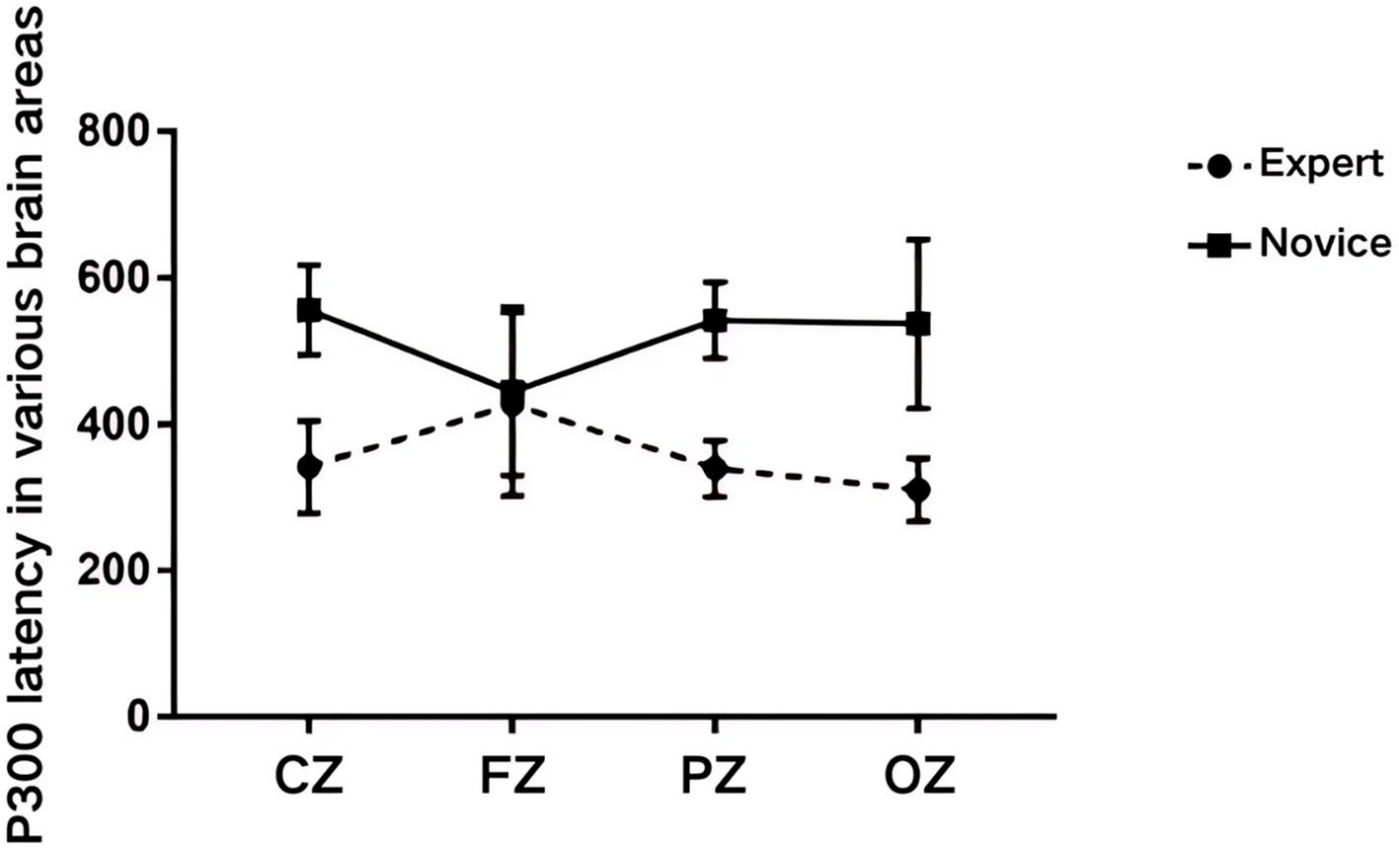
Differences in P300 components in different brain regions in offside situations.

The primary effect of group was significant, *F*(1,16) = 73.58, *p* < 0.001, *η*^2^ = 0.82. The main effect of different brain regions was not significant [*F*(3,48) = 0.28, *p* = 0.84 > 0.05, *η*^2^ = 0.02]. The interaction between brain region and group was significant [*F*(3,48) = 6.25, *p* < 0.001, *η*^2^ = 0.281]. A simple effects analysis revealed a significant main effect of the central zone (CZ) *F*(1,16) = 53.73, *p* < 0.001, *η*^2^ = 0.77. The main effect of the frontal zone (FZ) was not significant, *F*(1,16) = 0.09, *p* = 0.76 > 0.05, *η*^2^ = 0.01. The main effect of the parietal zone (PZ) was significant, *F*(1,16) = 88.00, *p* < 0.001, *η*^2^ = 0.85. The main effect of the occipital zone (OZ) was significant, *F*(1,16) = 31.00, *p* < 0.001, *η*^2^ = 0.66. As shown in Figure 4, the latencies of the P300 components in CZ, PZ, and OZ were significantly shorter in the expert panel than those in the novice group.

A 2 × 2 × 4 three-way repeated measures ANOVA with P300 wave amplitude as the dependent variable, group, the four electrode locations in the brain region, and stimulus type as the independent variable showed a significant main effect of the brain region [*F*(3,48) = 3.45, *p* = 0.024 < 0.05, *η*^2^ = 0.18], with significant differences in the amplitude of the early component P300 across different brain regions and a significant interaction effect between brain region and stimulus type, *F*(3,48) = 3.33, *p* = 0.03 < 0.05, *η*^2^ = 0.17; Due to the significant interaction effect, further analysis of simple effects revealed that the amplitude of the P300 wave generated in the non-offside condition was significantly larger than that in the offside condition. Its amplitude reached a maximum in the occipital region.

#### 3.3.2 Late slow wave

The late slow wave is the brain’s further information processing of the experimental task, including the process of feeling, perception, memory, analysis, and judgment of the experimental material. According to the purpose of the experiment, the cerebral cortex was divided into five main regions: prefrontal, frontal, central, parietal, and occipital. In each region, three representative electrodes were selected from the left and right hemispheres, and the resulting values were averaged across brain regions. The regions included the left and right prefrontal, frontal, central, parietal, and occipital regions. A repeated-measures ANOVA was performed with the five brain regions as independent variables and the mean wave amplitude as the dependent variable for the occurrence of offside images.

No significant differences were found in the prefrontal, frontal, and central regions; however, significant differences were observed in the parietal and occipital regions. A repeated-measures ANOVA was performed with the mean amplitude of the left and right parietal regions as the dependent variable, and group and left and right brain regions as independent variables. The main effect of the left and right brain regions was not significant [*F*(1, 18) = 3.35, *p* = 0.08 > 0.05, *η*^2^ = 0.16]. The main effect of group was significant, *F*(1,18) = 8.61, *p* < 0.001, *η*^2^ = 0.32. The interaction effect was significant, *F*(1,18) = 9.24, *p* < 0.001, *η*^2^ = 0.34. A simple effects analysis revealed that the mean amplitude of the expert group was significantly higher than that of the novice group in the left parietal region (experts: 1.91 ± 2.21; novices: −0.78 ± 0.79) and that the mean amplitude of the expert group was significantly lower than that of the novice group in the right parietal region (experts: 2.37 ± 0.72; novices: 1.13 ± 1.66).

Again, a repeated-measures ANOVA was performed with the mean amplitude of the left and right occipital regions as the dependent variable, and group and left and right occipital regions as independent variables. The main effect of the left and right occipital regions was not significant, *F*(1,18) = 0.02, *p* = 0.89 > 0.05, *η*^2^ = 0.01. The main effect of group was not significant, *F*(1,18) = 0.24, *p* = 0.63 > 0.05, *η*^2^ = 0.01. However, the interaction of the left and right occipital regions with the group was significant [*F*(1, 18) = 9.83, *p* < 0.001, *η*^2^ = 0.35]. Simple effect analyses revealed that in the right brain region, the mean wave amplitude was significantly lower in the expert group (0.24 ± 1.85) than in the novice group (1.67 ± 0.87). In the left-brain region, the mean wave amplitude was significantly higher in the expert group (1.47 ± 0.79) than in the novice group (0.55 ± 1.75) for the subjects in the expert group. The mean amplitude of the occipital region was significantly higher on the left side (1.47 ± 0.79) than on the right side (0.24 ± 1.85) in the expert group, but the difference in the novice group was marginally significant (*p* = 0.06 [CI: −0.05 to 2.29]) and was lower on the left side (0.55 ± 1.75) than that on the right side (1.67 ± 0.87).

#### 3.3.3 Decision stage ERP components

**Figure 5:**
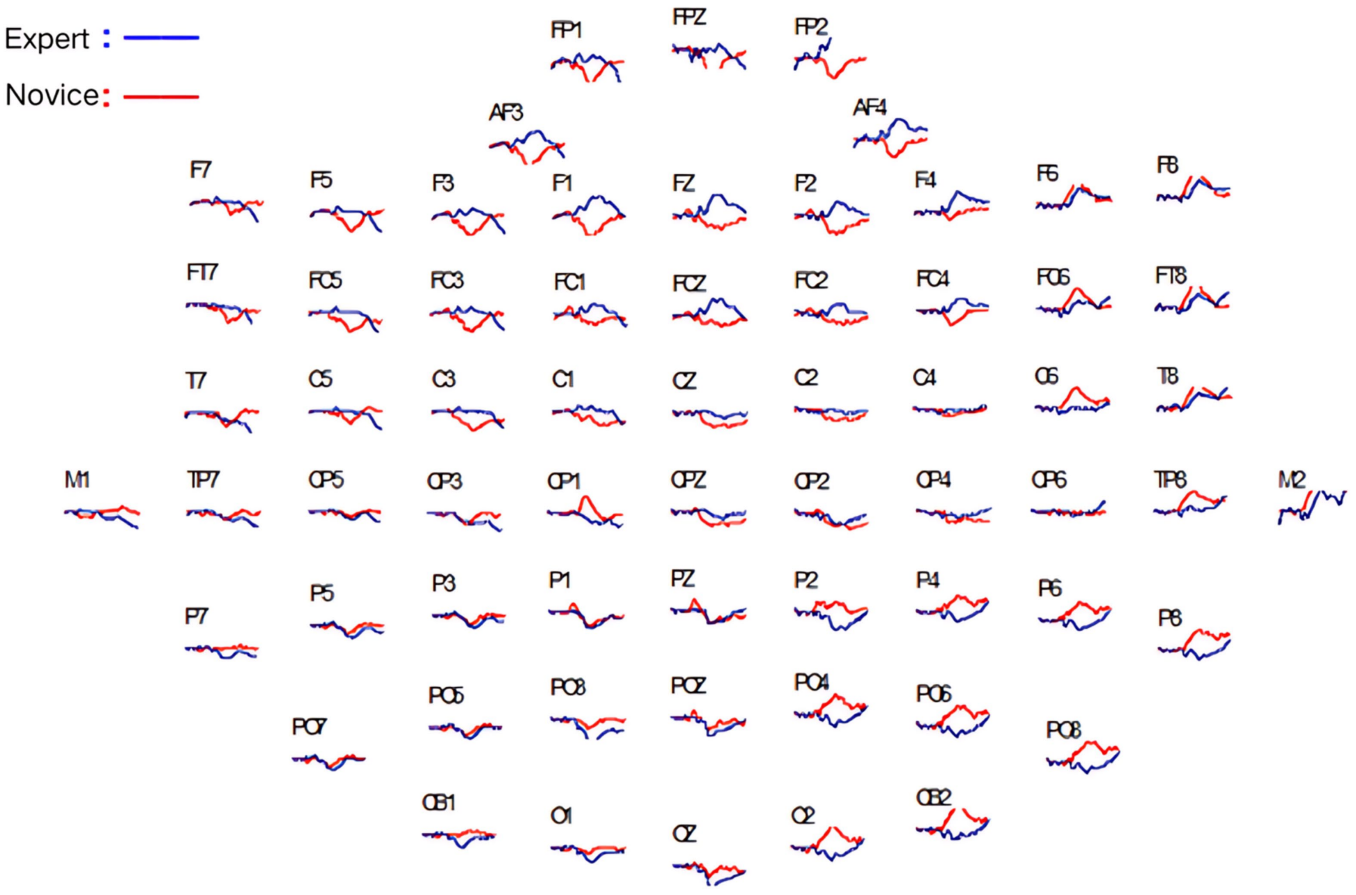
Grand-average amplitude waveform during the response-locked decision-making stage.

**Figure 6:**
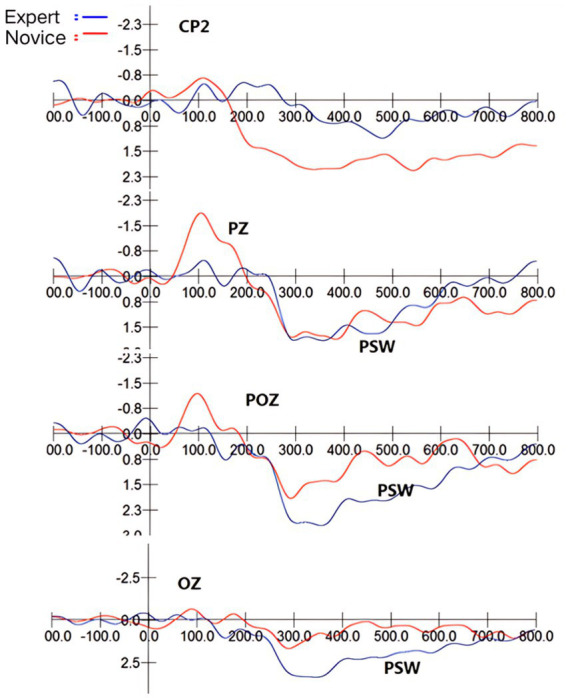
PSW component topography at selected electrode sites during the decision-making stage.

Based on the participants’ button press responses to different images and according to the overall average representation of this segmentation process, as shown in Figure 5, as well as the comparison representations of the electrode points in the midline section, the observation of the superimposed ERP waveform plots of all participants showed that after the occurrence of the P300 component of the participant, a pronounced late slow positive wave occurred approximately 450-500 ms after the participant’s response. This component can be identified as a PSW component. As the experiment was a subject-response task, this component could be related to the subject’s recognition of the target stimulus and decision-making process. As shown in Figure 6, a significant PSW component was evoked in the frontal, central, and occipital areas. Repeated-measures ANOVA was performed using the FZ, CZ, PZ, and OZ electrode point amplitudes as dependent variables, with group, stimulus type, and brain region as independent variables. The results are shown in. The effect of brain region and group interaction was significant [*F*(3, 48) = 4.62, *p* = 0.01 < 0.05, *η*^2^ = 0.44]. Another simple effect analysis showed that the frontal (FZ: 2.58 ± 0.94) and occipital (OZ: 2.67 ± 0.84) amplitudes of the participants in the expert group were significantly higher than those of the other parietal regions (PZ: 1.54 ± 0.46) and the central region (CZ: 1.85 ± 0.72), while the novice group showed no difference (Figures 5, 6).

Furthermore, a repeated-measures ANOVA showed no significant effect on the latency of this component.

## 4 Discussion

### 4.1 Spatial working memory extraction features for offside decisions by assistant referees

The results showed that reaction time differed significantly in the expert group. Experts made faster decisions for offside images than for non-offside images. In the novice group, reaction time did not differ between the two stimulus types. Experts also responded faster than novices for offside images. The group difference was smaller for non-offside images. These results indicate that experienced assistant referees performed better in offside decisions. In many high-pressure sports, athletes need to predict opponents’ movements and ball trajectory in advance (Lorains et al., 2013). Referees also need to integrate several cues quickly and intuitively during foul judgments (Plessner et al., 2023). Previous research suggests that people can retrieve useful knowledge from memory when they face realistic sport situations (Mann et al., 2007). Therefore, task performance depends on the ability to retrieve and use perceptual information. This ability is based on memory structures formed from experience. As experience increases, more examples are stored in the internal retrieval system. This can improve task accuracy and form an expertise effect. For example, FIFA assistant referees were more accurate than Belgian assistant referees in offside decisions (76.4% vs. 67.5%; Gilis et al., 2008).

For this study, two groups with different levels of refereeing skill were selected: expert and novice groups. Not only did the expert group have more refereeing experience than the novice group, but they also had more match practice and therefore had more instances of relevant knowledge experiences stored in their memory systems. During the experimental process, when the stimulus was presented for a short period, the participants in the expert group were able to quickly retrieve task-specific offside information from their mental instance memory according to the stimulus characteristics and respond appropriately. However, the participants in the novice group, due to the lack of relevant instances stored in their memory system, had longer reaction times when responding to the stimulus, were less accurate, and their task processing during completion, which relied mainly on the presented scene information, was insufficient. Theoretically, comparing novices with experts in the same field can deepen our understanding of the perceptual information-processing abilities of key decision-makers (Fortin-Guichard et al., 2020).

### 4.2 Visual search characteristics of assistant referees’ offside call decisions

Visual search is important when soccer referees make foul decisions. It helps them collect task-related information. However, visual search in excellent on-field decision making is still not fully understood (van Biemen et al., 2023b). In the present study, novices showed more fixation points within the area of interest than expert assistant referees. This suggests that expert referees searched information more efficiently. Good sport performance requires an accurate search for important information. It also requires ignoring irrelevant or distracting information and shifting attention quickly to key cues (Ziv et al., 2020). Similar results have been reported in cricket, rugby, and basketball referees. Experts usually show stable and precise gaze behavior. They can capture global information quickly and focus on important information (Ramachandran et al., 2021; Moore et al., 2019; Ruiz et al., 2023). By contrast, novices showed weaker overall spatial localization. Their gaze points were more scattered in the area of interest. In the expert group, average gaze time did not differ between offside and non-offside stimuli. In the novice group, average gaze time was shorter in offside situations than in non-offside situations. For non-offside judgments, novices had longer mean gaze duration than experts. This is consistent with Moreira et al. (2020). Shorter gaze duration may indicate that expert referees perceive relevant cues more quickly. The novice group was more affected by stimulus type. The expert group was less affected by the two situations. The eye-tracking maps also showed that both groups mainly looked at overlapping areas of interest, especially the ball and the offside line.

Decision accuracy is higher when assistant referees focus on the offside line at the time of the pass. This is true across expertise levels (Schnyder et al., 2017). Catteeuw et al. (2009b) also found that assistant referees fixate on the offside line before, during, and after a pass. Their gaze does not clearly shift from the passer to the receiver. Therefore, the offside line and related spatial cues may provide an important basis for offside decisions. The present study showed that expert assistant referees used more efficient visual search sequences. For experts, the player position, ball position, and spatial relation in the image may not be the only cues for the decision (Plessner and Betsch, 2002). For novices, these cues formed the main basis for judgment. Experts mainly focused on the relationship between the offside line, the ball, and the player in possession. They could retrieve similar examples from memory. They also matched these examples with the current spatial relation. Thus, experienced referees processed information in a more top-down way. Novices relied more on the ball, the passer, and the presented spatial relation. Their processing was more bottom-up.

### 4.3 Neural mechanisms in the brain of assistant referees’ offside call decisions

Observation and analysis of the ERP overall mean curve plots for experts and novices in offside and non-offside situations revealed that both groups of referees with different hierarchical levels evoked different levels of activation of the N2 and P300 components in different brain regions early in the stimulus presentation. Related studies have shown (Jolicœur et al., 2008) that the evocation of N2 is mainly associated with the correct recognition of the target and that this component is considered as the process of recognition, detection, formation, and formulation of the stimulus signal strategies and the conscious monitoring and controlling behavior before or during the behavior, processing and adjusting the feedback information about the outcome of the behavior after the behavior has occurred. Adaptation process. Because the experiment was a stimulus-presentation type, N2 mainly reflected the attentional quality of the visual cortex during the processing of visual stimuli. It has been shown (Jolicœur et al., 2008) that the N2 component in the posterior part of the brain is primarily associated with visual search tasks, and this component has been widely identified as a marker of whether attention capture in the brain is in the visual system. The amplitude of the N2 wave tends to be related to the attentional area, and the depth of processing is proportional to the extreme negativity, leading participants to generate greater attentional resources. The N2 component is considered an important indicator of cognitive control and is often used in decision-making.

The latency of the N2 component was later in the central, parietal, and occipital areas in the novice group than in the expert group, suggesting that novices require more time to fully capture attention compared to experts. This is because novice referees have lower cognitive matching and cognitive control than experts in new game situations. Experts have a cognitive advantage due to the accumulation of expertise and training over time, which is consistent with empirical theory (Schaefer and Scornaienchi, 2020). After several years of training, experienced referees have a much greater knowledge base and refereeing experience than inexperienced referees. As an inexperienced referee, the graphical representations of the attacking and defending teams and the positional relationship of the ball are not sufficiently visualized in their memory. As inexperienced referees, they had unclear memories of the graphical representations of the attacking and defending sides, and the positional relationship of the ball was not clear enough, which made it difficult for them to recall and recognize the stimulus information and made their memory matching ineffective. Their attention capture is less precise than that of the experts. Because of their extensive knowledge and experience reserves, the expert group was able to quickly match the stimulus to the experience model in their minds upon receiving it. The matching speed was high, giving them enough time to distinguish between the real stimulus features and the experience model, adapt them to the level of cognitive assessment, and adjust in time faster than the subjects in the novice group. Because inexperienced judges have little practical experience, group novices have a lower experience threshold for task difficulty when given the same stimulus images, whereas the experience of experts ensures that their judgments are accurate and quick. Thus, the expert group had an earlier latency, confirming these results.

The P300 component is typically evoked during low-probability events in the oddball paradigm. The P300 component appears as a positive wave that occurs 300–650 ms after stimulus onset and is the third positive component produced in nociceptors during the stimulus perception phase (van Biemen et al., 2023b). The P300 is believed to reflect stimulus evaluation, and its latency correlates with the time required for stimulus detection, pattern recognition, classification, and memory template matching (Gibbons, 2022). This component reflects neural activity during cognitive processing and content updating at the completion or end of information processing. Thus, the P300 latency can indirectly indicate the continuity in the completion of the recognition and processing of the stimulus, which objectively determines the classification and encoding of external stimuli by the brain and the degree of recognition of fast and slow stimuli, that is, the required time, assessed by the human brain, to carry out the process of evaluating external stimuli (Kida et al., 2023). In other words, when the stimulus appears to the target during collection and processing, it determines the size of the latency. Conversely, its amplitude indicates the mental resources the individual invests in this process and the difficulty of the task. The information provided by the current environment is accepted by the mind and then represented in the brain’s information base, that is, in memory content. After receiving a new stimulus, the old representation influences the cognitive processing of the new stimulus, and both are integrated into the original representation to form a continuously updated new representation. The P300 component is generated during the display-updating process.

The P300 amplitude should be interpreted cautiously. In the present oddball task, the lower P300 amplitude in experts was not interpreted as lower task relevance. It was interpreted as a lower additional demand for cognitive resources after the offside configuration had been evaluated. P300 latency is often related to stimulus evaluation, pattern recognition, classification, and memory-template matching. P300 amplitude is related to attentional resource allocation and task demand. Thus, the larger P300 amplitude in novices may reflect more effortful evaluation of the presented scene information, especially in parieto-occipital sites. However, P300 amplitude is also affected by stimulus probability, task relevance, and motivation. Therefore, this result should be considered together with the behavioral and eye-movement findings.

In the present study, the greatly increased P300 amplitudes of novice football assistant referees were observed in the parieto-occipital region of the brain and varied with stimulus material, with larger amplitudes evoked in the non-offside condition. More specifically, spatial working memory was involved in the cognitive and decision-making processes underlying offside calls between the participants in the expert and novice groups, and the parieto-occipital lobe played an important role in stimulus search. The novice group referees, due to their limited participation in the game and lack of experience in offside decision-making, lacked sufficient knowledge of the overall field of play, and did not have a deep enough spatial working memory for the offside situation that occurred. Therefore, their final processing was more in-depth than that of the expert group, thus inducing a greater P300 amplitude. At the same time, it also demonstrates the existence of a specialized advantage in the sports domain, as the expert group’s years of training have led to a unique specialized visuospatial working memory system; thus, the expert group did not lead to deeper recognition and assessment. This is consistent with the conclusions of other studies. It has been shown that the decision speed of experts depends, to some extent, on the familiarity of spatial environmental memory in long-term memory (Ashford et al., 2021; Ramanayaka et al., 2023). Just as there is variability in the spatial working memory capacity for chess between grandmaster chess experts and general chess experts, grandmaster experts perform much better than general chess experts in predicting chess games (Smith et al., 2021).

Overall, the N2 and P300 results provide preliminary ERP evidence. Expert assistant referees may need fewer additional cognitive resources at the early stage of stimulus presentation. This may be related to their specialized visuospatial working memory system. This explanation is consistent with the behavioral and eye-movement findings. However, it should be treated with caution because participant-level artifact-free trial counts could not be reconstructed.

By statistically analyzing the average wave peaks of the subjects’ late slow waves during epochs from 1,000 to 2,500 ms after stimulus presentation, it was found that experts and novices differed in the parietal and occipital areas. The difference between the mean amplitudes of the left and right occipital regions in the expert group and the decision-making speed of the expert group depended, to some extent, on the degree of familiarity with spatial environment memory in long-term memory. The mean amplitude of the left occipital region in the expert group was significantly higher than that of the right occipital region. However, the difference in the novice group was borderline significant, and the left occipital region had a lower mean amplitude than the right. This result is consistent with the findings of Costa et al.'s (2023) study, in which all groups showed that the left hemisphere had a greater amplitude than the right hemisphere, and the mean amplitude of the novice group was greater than that of the expert group. The mean amplitude of the expert was the smallest. Some scholars have proposed the theory of unilateralization of the functions of the two hemispheres of the brain through their studies (Bayne, 2008), which suggests that there is a difference in the processing of information between the left and right hemispheres of the brain, and that the left hemisphere processes information in a fragmented, analytical, and sequential manner, while the right hemisphere processes information in an intuitive, finished, and integrated manner.

Nessler et al. (2006), who found a similar ERP component in an event-related potential study of semantic memory processes, suggested that the increase in the amplitude of the late component evoked in the frontal regions is importantly related to the process of task forgetting during retrieval. This component may be related to the processing of analogous mappings between concepts. The late component reflects the process of stimulus recognition after consciousness. Furthermore, some scientists have argued (Nakamoto and Mori, 2008) that the late component is induced during false beliefs, conditional reasoning, etc., and that similar scalp slow-wave potentials are induced during operational tasks such as recognition and information storage that are required in the working memory. Late positive wave amplitudes showed significant differences mainly in the frontal and central frontal regions, and such late-sustained positive potentials reflect the number of attentional resources and the degree of motivational involvement that the individual invests in the image-recognition task. Based on the average waveform plots presented in Figure 5 of the text, the parietal and occipital regions of the late period of the time course had a persistent positive component. In cognitive psychology, the prefrontal lobe plays a role in the temporary storage, encoding, activation, control, and coordination of information in working memory, and a decrease in P300 waveform amplitude reflects that fewer resources are currently allocated to the current task (Yang et al., 2008). The novice group showed a large negative deviation in the prefrontal area compared with the expert group, suggesting that novices reason analogously, relying on partial information from the playing field, and must invest greater cognitive resources during this time interval to complete their judgment. Conversely, the expert group does not need to invest even a small number of cognitive resources in motivational involvement (Song et al., 2007; Radlo et al., 2001).

In the present experiment, both groups showed a prolonged positive wave after the button-press response. This wave appeared after the P3 component, about 450 ms after the response, and continued to about 750 ms. Therefore, it was identified as a PSW. The PSW is a late, slow positive wave. It usually appears after cognitive operation and stimulus evaluation. In this study, both groups showed clear polarity deflection in the anterior brain region (precentral area), as shown in Figure 6. No significant latency effect was found. However, the interaction between the brain region and group was significant. In the expert group, frontal and occipital amplitudes were higher than parietal and central amplitudes. In the novice group, no such difference was found. These findings suggest that PSW may reflect higher cognitive processes during offside decisions. These processes may include self-evaluation and working-memory updating. Previous studies also suggest that PSW may reflect the continuous updating of working memory (Radlo et al., 2001).

In most ball sports, working memory is closely related to predicting opponents’ actions. Previous studies show that working-memory encoding is related to neuroelectric activity in the medial prefrontal cortex. The prefrontal cortex is an important physiological basis for working-memory encoding. The polarity deviation in the medial prefrontal area in this study is consistent with this view. Experts have accumulated extensive experience over many years of offside decision-making practice. Compared with inexperienced referees, they integrate perception, cognition, and judgment more effectively. This may be related to efficient task-related neural networks (Chen et al., 2022). Their stored offside experiences may support accurate and smooth top-down processing of current stimuli. In this study, only pictorial stimuli were used. Future studies should examine whether similar results can be found when referees judge more dynamic offside situations. Furthermore, the late slow wave spanned a long time window. Future studies may divide it into smaller time windows to compare neural mechanisms between experts and novices (Catteeuw et al., 2010b).

### 4.4 Limitations

First, the sample size was limited. The behavioral and eye-movement analyses included 26 assistant referees. The ERP analyses included 18 participants after excluding EEG recordings with excessive drift. Therefore, the findings should be generalized cautiously. The expert group mainly came from Chinese professional leagues. The novice group consisted of lower-level assistant referees with limited experience. Thus, the findings may not represent referees from other countries, competition levels, or demographic groups. Age and officiating experience were also confounded. The expert group was older than the novice group. Because age was not independently controlled, the observed differences should be interpreted as expertise-associated differences. They should not be regarded as definitive causal effects of experience alone. Future studies should use larger, more diverse, and preferably age-matched samples. Women officials and referees from different regions and levels should also be included.

Second, the ERP trial structure should be taken into account when interpreting the neural findings. The offside condition had a maximum of 50 trials per participant before artifact rejection. The non-offside condition had a maximum of 150 trials. This imbalance was part of the oddball design. It may have introduced expectancy or stimulus-probability effects, especially for P300 amplitude. The original preprocessing logs were not available. Therefore, we could not accurately reconstruct the number of artifact-free trials for each participant and condition. This limits the evaluation of participant-level ERP data quality. It may also reduce the reliability of ERP averages, especially for the offside condition and later components such as P300 and PSW. Therefore, the ERP results should be regarded as preliminary evidence. They should be used to complement the behavioral and eye-movement findings. Future studies should increase the number of target trials when possible. They should also consider more balanced stimulus sets and prospectively report counts of retained trials, rejection rates, and other ERP data-quality indices.

Third, the stimulus materials were static images rather than continuous match sequences. This design helped control ERP recording and compare offside with non-offside situations. However, it removed several cues used in live matches. These cues include player movement, ball trajectory, passing timing, and changing tactical context. Therefore, the present findings may not fully reflect visual search and neural processing during real-time officiating (van Biemen et al., 2023b). Future studies should use short video clips, immersive virtual reality, or field-based wearable eye tracking. These methods can test whether the present expertise-related patterns also appear in more representative decision-making environments. For example, van Biemen et al. (2023a) found that football referees’ visual-motor behavior in VR was similar to on-field behavior. Their behavior in VR also differed from standard video viewing. This supports the value of more representative stimuli in future officiating research.

Finally, the present study compared only the temporal characteristics of different brain regions without retrospective analysis. Subsequent studies may be more informative if they combine ERP with complementary methods such as fMRI, functional network analysis, or structural network analysis.

## 5 Conclusion

Expert assistant referees showed faster reaction speed and higher offside decision accuracy than novice assistant referees. This supports the existence of an expertise advantage in offside decisions in soccer. This advantage may be related to rich in-field refereeing experience and a unique visual search strategy. The novice group showed a local processing bias in visuospatial working memory. This made it difficult for them to adopt a block search strategy. The behavioral and eye-movement findings, together with preliminary ERP evidence, suggest that experts may have more efficient memory matching, attention capture, and cognitive-resource allocation. Expert referees may need fewer additional cognitive resources in the early stage of stimulus presentation. This helps them capture attention faster and complete the identification and encoding of decision-related information. Because the original ERP preprocessing logs could not be retrieved, the neural findings should be interpreted cautiously. Future studies should verify them with artifact-free retained trial counts for each participant and condition.

## Data Availability

The raw data supporting the conclusions of this article will be made available by the authors, without undue reservation.
